# Optimal designs for copula models

**DOI:** 10.1080/02331888.2015.1111892

**Published:** 2016-01-08

**Authors:** E. Perrone, W.G. Müller

**Affiliations:** ^a^ Department of Applied Statistics, Johannes Kepler University Linz, 4040Linz, Austria

**Keywords:** copulas, design measure, Fisher information, stochastic dependence, clinical trials, 62K05

## Abstract

Copula modelling has in the past decade become a standard tool in many areas of applied statistics. However, a largely neglected aspect concerns the design of related experiments. Particularly the issue of whether the estimation of copula parameters can be enhanced by optimizing experimental conditions and how robust all the parameter estimates for the model are with respect to the type of copula employed. In this paper an equivalence theorem for (bivariate) copula models is provided that allows formulation of efficient design algorithms and quick checks of whether designs are optimal or at least efficient. Some examples illustrate that in practical situations considerable gains in design efficiency can be achieved. A natural comparison between different copula models with respect to design efficiency is provided as well.

## 1. Introduction

Due to their flexibility in describing dependencies and the possibility of separating marginal and joint effects copula models have become a popular device for coping with multivariate data. in many areas of applied statistics eg. for insurances,[1] econometrics,[2] medicine,[3] marketing,[4] spatial extreme events,[5] time series analysis,[6] even sports [7] and particularly in finance.[8]

The concept of copulas, however, has only been rarely employed in experimental design with notable exceptions of spatial design in [9,10], and sequential trials in [11]. The design question for copula parameter estimation has to our knowledge just been raised in [12], where a brute-force simulated annealing optimization was employed for the solution of a specific problem. By this paper we provide the necessary theory for fully embedding the situation into optimal design theory. Particularly we provide a Kiefer–Wolfowitz type equivalence theorem [13] in Section 5 as a basis for a substantial analysis of the arising issues in the example sections.

To be more concrete, let us consider a vector $x^{T}=(x_{1},\ldots ,x_{r})\in X$ of control variables, where $X\subset R^{r}$ is a compact set. The results of the observations and of the expectations in a regression experiments are the vectors:

$$\begin{array}{rl} y(x) & =(y_{1}(x),\ldots ,y_{m}(x)),\\ E[Y(x)] & =E[(Y_{1},\ldots ,Y_{m})]=\eta (x,\beta )=(\eta_{1}(x,\beta ),\ldots ,\eta_{m}(x,\beta )), \end{array}$$

where $\beta =(\beta_{1},\ldots ,\beta_{k})$ is a certain unknown vector of marginal parameters to be estimated and $\eta_{i}\;(i=1,\ldots ,m)$ are known functions. Let us call $F_{Y_{i}}(y_{i}(x,\beta ))$ the marginal cumulative distributions of each $Y_{i}$ for all i=1,…,m and $f_{Y}(y(x,\beta ),\alpha )$ the joint probability density function of the random vector Y, where $\alpha =(\alpha_{1},\ldots ,\alpha_{l})$ are unknown (copula) parameters. In the remainder of the paper we will focus on the case m=2, but generalizations of our results are possible.

Definition 2. Let I=[0,1]. A *two-dimensional copula* (or *2-copula*) is a bivariate function C:I×I⟶I with the following properties:

1. for every $u_{1},$ $u_{2}\in I$
  (1) $$C(u_{1},0)=0,\;C(u_{1},1)=u_{1},\;C(0,u_{2})=0,\;C(1,u_{2})=u_{2};$$
2. for every $u_{1},$ $u_{2},$ $u_{3},$ $u_{4}\in I$ such that $u_{1}\leq u_{3}$ and $u_{2}\leq u_{4},$
  $$C(u_{3},u_{4})-C(u_{3},u_{2})-C(u_{1},u_{4})+C(u_{1},u_{2})\geq 0.$$

Now let $F_{Y}$ be a joint cumulative distribution function (cdf) with marginal cdfs $F_{Y_{1}}$ and $F_{Y_{2}}$. According to Sklar's theorem [14] there exists then a 2-copula *C* such that

(2) $$F_{Y}(y_{1},y_{2})=C(F_{Y_{1}}(y_{1}),F_{Y_{2}}(y_{2}))$$

for all reals $y_{1}$, $y_{2}$. If $F_{Y_{1}}$ and $F_{Y_{2}}$ are continuous, then *C* is unique; otherwise, *C* is uniquely defined on $\mathrm{Ran}(F_{Y_{1}})\times \mathrm{Ran}(F_{Y_{2}})$. Conversely, if *C* is a 2-copula and $F_{Y_{1}}$ and $F_{Y_{2}}$ are distribution functions, then the function $F_{Y}$ given by Equation (2) is a joint distribution with marginals $F_{Y_{1}}$ and $F_{Y_{2}}$.

## 2. Design issues

We need to quantify the amount of information on both (trend and copula) sets of parameters *α* and *β* respectively from the regression experiment embodied in the Fisher information matrix, which for an elemental information at a particular control *x* in the sense of Atkinson et al. [15] is a (k+l)×(k+l) matrix defined as

(3) $$m(x,\beta ,\alpha )=\left(\begin{array}{cc} m_{\beta \beta }(x) & m_{\beta \alpha }(x)\\ m_{\beta \alpha }^{T}(x) & m_{\alpha \alpha }(x) \end{array}\right),$$

where the submatrix $m_{\beta \beta }(x)$ is the (k×k) matrix with the (i,j)th element defined as

(4) $$E\left(-\frac{\partial^{2}}{\partial \beta_{i}\partial \beta_{j}}\log[f_{Y}(y(x,\beta ),\alpha )]\right),$$

and the submatrices $m_{\beta \alpha }(x)$ (k×l) and $m_{\alpha \alpha }(x)$ (l×l) are defined accordingly. Here we model the dependence between $Y_{1}$ and $Y_{2}$ with a copula function $C_{\alpha }(F_{Y_{1}}(y_{1}(x,\beta )),F_{Y_{2}}(y_{2}(x,\beta )))$ and find the joint density of the random variables from

$$f_{Y}(y(x,\beta ),\alpha )=\frac{\partial^{2}}{\partial y_{1}\partial y_{2}}C_{\alpha }(F_{Y_{1}}(y_{1}(x,\beta )),F_{Y_{2}}(y_{2}(x,\beta )).$$

Definition 4. For a concrete (discrete) experiment with *N* independent observations at n≤N support points $x_{1},\ldots ,x_{n},$ the corresponding total *information matrix* is

$$M(\xi ,\beta ,\alpha )=N^{-1}\sum_{i=1}^{n}w_{i}m(x_{i},\beta ,\alpha ),\;\sum_{i=1}^{n}w_{i}=1,\;\xi =\left\{\begin{array}{ccc} x_{1} & \ldots & x_{n}\\ w_{1} & \ldots & w_{n} \end{array}\right\},$$

with so-called design weights $w_{i}$.

The aim of approximate optimal design theory is concerned with finding an optimal design measure $\xi^{*}(\beta ,\alpha )$, such that it maximizes some scalar function φ(M(ξ,β,α)), the so-called design criterion. In the following we will consider only *D-optimality*, that is, the criterion φ(M)=log⁡detM, if *M* is non-singular. There exist several well written monographs on optimal design theory and its application, but in this paper we follow mainly the style and notation of Silvey.[16]

## 3. Equivalence theory

The cornerstone of a theoretical investigation into optimal design is usually the formulation of a Kiefer–Wolfowitz type equivalence relation, which is given in the following theorem. It is a generalized version of a theorem given without proof in [17] and follows from a multivariate version of the basic theorem given in [16], its full proof can be found in the Appendix.

Theorem 3.1. Denote by $\left(\bar{\beta },\bar{\alpha }\right)$ fixed values (local guesses) for the parameter vector. Then, the following properties are equivalent:

- $\xi^{*}$ is D-optimal;
- $tr[M(\xi^{*},\bar{\beta },\bar{\alpha }{)}^{-1}m(x,\bar{\beta },\bar{\alpha })]\leq (k+l),$ ∀x∈X;
- $\xi^{*}$ minimize $\max_{x\in X}tr[M(\xi^{*},\bar{\beta },\bar{\alpha }{)}^{-1}m(x,\bar{\beta },\bar{\alpha })],$ over all ξ∈Ξ.

This theorem provides simple checks for D-optimality through the maxima of

$$d(x,\xi^{*})=\mathrm{tr}[M(\xi^{*},\bar{\beta },\bar{\alpha }{)}^{-1}m(x,\bar{\beta },\bar{\alpha })],$$

which is usually called *sensitivity function*. It also allows us the use of standard design algorithms such as of the Fedorov-Wynn-type,[18,19] which will yield an optimal approximate design $\xi^{*}=\left\{\begin{array}{ccc} {x_{1}}^{*} & \ldots & {x_{n}}^{*}\\ w_{1}^{*} & \ldots & w_{n}^{*} \end{array}\right\}$.

Note that these resulting optimal designs will now depend not only upon the marginal model structure, but also upon the chosen copula and through the induced nonlinearities potentially also on the unknown parameter values for *α* and *β*, which is why we are resorting to localized designs around the values $\left(\bar{\beta },\bar{\alpha }\right)$. A sensitivity analysis with respect to the effect of these choice on a particular example can be found in [20].

Definition 6. For the comparison of designs define *D-Efficiency* of the design *ξ* with respect to the design $\xi^{*}$ as the ratio

(5) $${\left(\frac{|M(\xi ,\bar{\beta },\bar{\alpha })|}{|M(\xi^{*},\bar{\beta },\bar{\alpha })|}\right)}^{1/(k+l)},$$

where (k+l) is the number of the model parameters. We will report all our findings in percentage losses of these D-efficiencies.

## 4. Examples

A main question now of course concerns whether ignorance or wrong guesses of copula function and/or parameters may lead to inefficiencies of the designs.

### 4.1. Tools

For that purpose let us here give the list of copulas used in our examples (for more details see, eg. [21] or [22]). We provide the copula function along with the so-called Kendall's *τ*, which is a dependence measure that allows us to conveniently relate different copulas (for a definition and a more exhaustive comparison see [23]).

Definition 8.

1. *Product Copula*, which represents the independence case:
  $$C(u_{1},u_{2})=u_{1}u_{2},$$
  with τ=0.
2. *Gaussian Copula*:
  $$C_{\alpha }(u_{1},u_{2})=\frac{1}{2\Pi \sqrt{1-\alpha^{2}}}\int_{-\infty }^{\Phi^{-1}(u_{1})}\int_{-\infty }^{\Phi^{-1}(u_{2})}\exp\left(-\frac{{z_{1}}^{2}-2\alpha z_{1}z_{2}+{z_{2}}^{2}}{2(1-\alpha^{2})}\right)dz_{1}\;dz_{2},$$
  with α∈[−1,1] and τ=(2/Π)arcsin⁡(α).
3. *Farlie–Gumbel–Morgenstern (FGM)*:
  $$C_{\alpha }(u_{1},u_{2})=u_{1}u_{2}[1+\alpha (1-u_{1})(1-u_{2})],$$
  with α∈[−1,1] and $\tau =\frac{2}{9}\alpha$.
4. *Clayton*:
  $$C_{\alpha }(u_{1},u_{2})=[\max(u_{1}^{-\alpha }+u_{2}^{-\alpha }-1,0){]}^{-(1/\alpha )},$$
  with α∈(0,+∞) and τ=α/(α+2).
5. *Frank*:
  $$C_{\alpha }(u_{1},u_{2})=-\frac{1}{\alpha }\ln\left(1+\frac{\left(e^{-\alpha u_{1}}-1)(e^{-\alpha u_{2}}-1\right)}{e^{-\alpha }-1}\right),$$
  with α∈(−∞,+∞), and $\tau =1-(4/\alpha )(1-(1/\alpha )\int_{0}^{\alpha }(t/\left(e^{t}-1\right))\;dt)$.
6. *Gumbel*:
  $$C_{\alpha }(u_{1},u_{2})=\exp(-[(-\ln u_{1}{)}^{\alpha }+(-\ln u_{2}{)}^{\alpha }{]}^{1/\alpha }),$$
  with α∈[1,+∞) and τ=(α−1)/α.

### 4.2. The linear case

**Figure 1.**
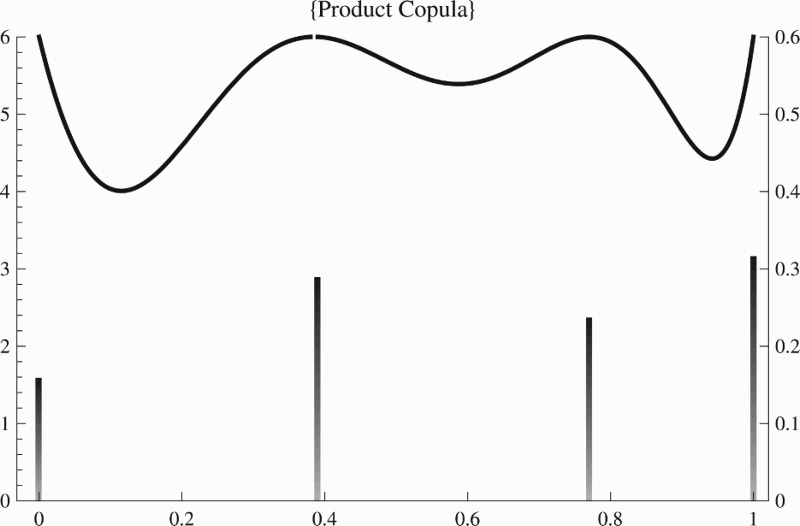
Sensitivity function (left axis) and optimal design (right axis) for the Fedorov example.

Let us first consider a simple example reported in [18]. For each design point x∈[0,1], we may observe an independent pair of random variables $Y_{1}$ and $Y_{2}$, such that

$$\begin{array}{rl} E[Y_{1}(x)] & =\beta_{1}+\beta_{2}x+\beta_{3}x^{2},\\ E[Y_{2}(x)] & =\beta_{4}x+\beta_{5}x^{3}+\beta_{6}x^{4}, \end{array}$$

which is linear in *β* and has dependence described by the product copula with Gaussian margins. Since this case is covered by Theorem 1, we were able to compute the optimal design $\xi^{*}$ by a standard algorithm and we display it in Figure 1 along with its sensitivity function. As rather typical $\xi^{*}$ is supported on only a small number (here four) of design points, smaller than the number of parameters. From the sensitivity function we can see that it is indeed optimum as it reaches (and not exceeds) the number of parameters at all design points. Furthermore our optimal design coincides with the one reported in [18], namely

(6) $$\xi^{*}=\left(\begin{array}{c} x_{i}^{*}\\ w_{i}^{*} \end{array}\right)=\left(\begin{array}{cccc} 0 & 0.38 & 0.76 & 1.0\\ 0.16 & 0.28 & 0.23 & 0.33 \end{array}\right).$$

Let us consider a more general case, for which the joint distribution is described by a Gaussian copula and we thus allow the random variables $Y_{1}$ and $Y_{2}$ to be dependent. In this case the joint probability function of the random vector $Y=(Y_{1},Y_{2})$ is simply

(7) $$\begin{array}{rl} F_{Y}(y_{1},y_{2}) & =C_{\alpha }(\Phi (y_{1}-\eta_{1}(x,\beta )),\Phi (y_{2}-\eta_{2}(x,\beta )))\\ & =\Phi_{2}(y_{1}-\eta_{1}(x,\beta ),y_{2}-\eta_{2}(x,\beta );\alpha ), \end{array}$$

where $\Phi_{2}(\cdot ,\cdot ;\alpha )$ denotes the bivariate normal cdf with correlation α∈(−1,1) and Φ denotes the cdf of the standard normal distribution N(0,1) (see [24]).

Our computations gave rise to the following

Corollary 4.2 For different values of *α* the optimal design is the same as for the independence case, which is the Gaussian case with α=0.

Note, that the sensitivity function now has a different scaling (with a maximum at 7) as we have an additional copula parameter. This corollary, however, is hardly surprising as this fact coincides with the classic findings for the multivariate Gaussian distribution by Krafft and Schaefer.[25]

But now for a contrast consider the FGM copula. Following our approach, we must calculate the density corresponding to the function:

$$\begin{array}{rl} C_{\alpha }(\Phi (Y_{1}(x;\beta )),\Phi (Y_{2}(x;\beta ))) & =\Phi (Y_{1}(x;\beta ))\Phi (Y_{2}(x;\beta ))\\ & \;\times [1+\alpha (1-\Phi (Y_{1}(x;\beta )))(1-\Phi (Y_{2}(x;\beta )))], \end{array}$$

which eventually leads to expressions like

$$E\left(-\frac{\partial^{2}}{\partial \beta_{i}\partial \beta_{j}}\log\left[\frac{\partial^{2}}{\partial y_{1}\partial y_{2}}C_{\alpha }(\Phi (Y_{1}(x;\beta )),\Phi (Y_{2}(x;\beta )))\right]\right)$$

for the information matrix. These integrals are not analytically solvable, but we can evaluate them numerically and we can use the algorithm in order to find the optimum designs.

Not surprisingly those optimal designs do depend upon the choice of $\bar{\alpha }$ (i.e. the assumed dependence) – and similar calculations can be performed for other copula functions as well. Some results are subsumed in Table 1, which displays the loss in D-efficiency that occurs by using the optimal design $\xi^{*}$ from Equation (6) compared to the respective optimal designs for various copula models and Kendall's *τ*. It can be seen that these losses are generally quite small for all considered copulas.

**Table 1.** Losses in D-efficiency (in bold) by ignoring the dependence in per cent.

	FGM		Clayton		Frank	
*τ*	$\bar{\alpha }$	D-eff	$\bar{\alpha }$	D-eff	$\bar{\alpha }$	D-eff
−0.15−	−0.67−	**0**.**29**	n.d.	–	−1.37	**0**.**10**
−0.10−	−0.45−	**0**.**23**	n.d.	–	−0.90	**0**.**10**
−0.05−	−0.22−	**0**.**59**	n.d.	–	−0.45	**0**.**10**
0.05	0.22	**0**.**68**	0.10	**0**.**16**	0.45	**0**.**10**
0.10	0.45	**0**.**39**	0.22	**0**.**13**	0.90	**0**.**10**
0.15	0.67	**0**.**28**	0.35	**0**.**34**	1.37	**0**.**10**
0.35	n.d.	–	1.08	**0**.**11**	3.51	**0**.**11**
0.75	n.d.	–	6.00	**0**.**27**	14.13	**0**.**16**

### 4.3. A binary bivariate model

In order to better investigate the role of the copula parameter, we analyse a more elaborate example with potential applications in clinical trials. Let us formally introduce the model. We consider a bivariate binary response $\left(Y_{i1},Y_{i2}\right)$, i=1,…,n with four possible outcomes {(0,0),(0,1),(1,0),(1,1)} where 1 usually represents a success and 0 a failure (of eg. a drug treatment). For a single observation denote the joint probabilities of $Y_{1}$ and $Y_{2}$ by $p_{y_{1},y_{2}}=\mathrm{pr}(Y_{1}=y_{1},Y_{2}=y_{2})$ for $\left(y_{1},y_{2}=0,1\right)$. In a clinical trial context $Y_{1}$ and $Y_{2}$ could represent efficacy and toxicity of a tested drug.

Now, define

(8) $$\begin{array}{rl} p_{11} & =C_{\alpha }(\pi_{1},\pi_{2}),\;p_{10}=\pi_{1}-p_{11},\\ p_{01} & =\pi_{2}-p_{11},\;p_{00}=1-\pi_{1}-\pi_{2}+p_{11}. \end{array}$$

The complete log-likelihood for the bivariate binary model is then given by

(9) $$l(\theta ;y)=\sum_{i=1}^{n}w_{i}l_{i}(\theta ;y),\;\theta =(\beta_{1},\beta_{2},\alpha ),$$

where $\beta_{1}$ and $\beta_{2}$ are the parameters associated with the respective margins and the log-likelihood for a single observation is given by

(10) $$l_{i}(\theta ;y)=y_{1}y_{2}\log p_{11}+y_{1}(1-y_{2})\log p_{10}+(1-y_{1})y_{2}\log p_{01}+(1-y_{1})(1-y_{2})\log p_{00}.$$

As shown in [26] the Fisher information matrix for a single observation can then be written as

(11) $$M(\theta ,\xi_{i})={\frac{\partial p}{\partial \theta }}^{T}\left(P^{-1}+\frac{1}{1-p_{11}-p_{10}-p_{01}}ee^{T}\right)\frac{\partial p}{\partial \theta },$$

where $p=(p_{11},p_{10},p_{01})$, P=diag(p) and $e=(1,1,1{)}^{T}$. Some useful formulae for calculating information matrices in copula models can also be found in [27].

A particular case of the introduced model has already been analysed in [17]. In that work, the authors assume the marginal probabilities of success given by the models

(12) $$\log\left(\frac{\pi_{i}}{1-\pi_{i}}\right)=\beta_{i1}+\beta_{i2}x,\;i=1,2$$

with x∈[0,10] and ‘localized’ parameters ${\bar{\beta }}_{1}=[-1,1]$ and ${\bar{\beta }}_{2}=[-2,0.5]$. The considered joint cdf is the Gumbel cdf, which corresponds to the following choice for the probability of success $p_{11}$:

$$p_{11}=F_{Y_{1},Y_{2}}(\pi_{1},\pi_{2})=\pi_{1}\pi_{2}(1+\alpha (1-\pi_{1})(1-\pi_{2})),$$

that is, in terms of copulas, the FGM copula.

In [17], the choice of this model is highlighted by arguing that it allows for dependence between efficacy and toxicity and it is claimed that including estimation of *α* rather then independently analysing efficacy and toxicity is preferable. We reanalyse this example studying both the role of the copula parameter and the impact of a particular type of dependence structure on the D-optimal designs obtained.

To get a clearer idea of the role played by the copula parameter, let us first focus on the benchmark case of independence, described both by the Product copula and the FGM copula with α=0. However, using these two copulas has substantially different interpretation and effect. In the one case (Product copula) we completely ignore potential dependence, whereas in the other case (FGM copula) we allow for its estimation, but assume it inexistent.

In a second step, to examine the impact of the dependence structure, we compare the model analysed in [17] with the more general ones proposed in [12]. Those have the same assumptions on the marginals probabilities as in [17], as well as same design space and initial parameter for the betas, but the dependencies are instead represented by using the copulas Frank, Gumbel, and Clayton. Note that in [12] the authors employed a brute-force simulated annealing algorithm for their calculations and had no means for checking definitive optimality, which is now possible through the equivalence theorem (Theorem 5.1) provided.

Now, using the D-optimal designs for the FGM copula and for the Product copula as benchmarks, we note the losses in D-efficiency in per cent as reported respectively in Table 2 (Product copula) and in Table 3 (FGM). In both cases, the losses are much stronger than in the previous example.

**Table 2.** Losses in D-efficiency (in bold) by ignoring the dependence in per cent (product copula).

	Frank		Clayton		Gumbel	
*τ*	$\bar{\alpha }$	D-eff	$\bar{\alpha }$	D-eff	$\bar{\alpha }$	D-eff
0.11	1.00	**1**.**72**	0.24	**1**.**75**	1.12	**0**.**95**
0.45	5.00	**1**.**31**	1.68	**1**.**49**	1.84	**1**.**29**
0.66	10.00	**1**.**87**	3.98	**0**.**71**	3.00	**2**.**31**
0.76	15.00	**2**.**89**	6.42	**2**.**84**	4.21	**2**.**99**
0.82	20.00	**3**.**10**	8.89	**9**.**48**	5.45	**3**.**25**

**Table 3.** Losses in D-efficiency (in bold) in per cent with respect to the FGM copula (α=0).

	Frank		Clayton		Gumbel	
*τ*	$\bar{\alpha }$	D-eff	$\bar{\alpha }$	D-eff	$\bar{\alpha }$	D-eff
0.11	1.00	**0**.**01**	0.24	**2**.**42**	1.12	**0**.**87**
0.45	5.00	**0**.**36**	1.68	**1**.**13**	1.84	**0**.**5**
0.66	10.00	**3**.**18**	3.98	**1**.**34**	3.00	**2**.**84**
0.76	15.00	**5**.**63**	6.42	**5**.**54**	4.21	**5**.**13**
0.82	20.00	**6**.**24**	8.89	**13**.**94**	5.45	**6**.**12**

Analysing these results by focusing on the Frank and the Gumbel copulas, one can notice that lower losses in Table 3 correspond to the lower values of *τ*. Conversely, the losses already become much higher in Table 3 for a moderate level of the association *τ*. Moreover, looking at the results for the Clayton copula, we even have lower losses by ignoring the dependence for almost all the levels of the association *τ*. This suggests, at first glance, that it is not generally preferable to insert a dependence parameter to be estimated, since this might increase the losses when the model is chosen badly.

Interestingly, if the copula parameter is not estimated, that is, if the model is just a four parameter model, the optimal designs found are almost the same for all the investigated copulas, and can be represented by

$$\xi^{*}=\left(\begin{array}{c} x_{i}^{*}\\ w_{i}^{*} \end{array}\right)=\left(\begin{array}{ccc} ∼0 & 2.80 & 6.79\\ 0.42 & 0.36 & 0.22 \end{array}\right).$$

**Figure 2.**
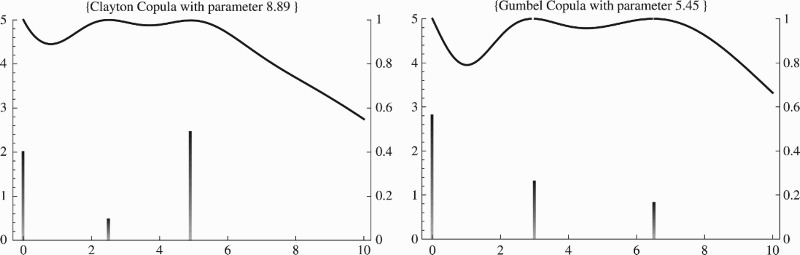
The optimal designs and the sensitivity functions for the binary example (Clayton left, Gumbel right). The copula parameters chosen correspond to Kendall's τ=0.816.

Evidently, the structure of the dependence has an impact as soon as its parameter requires estimation. In Figure 2 we display the designs and sensitivity functions for a representative case contrasting the very different optimal designs for a Clayton and a Gumbel copula with identical Kendall's *τ*. It will therefore be of great practical value to compare different copulas with respect to their optimal design properties (as well as eventually to be able to efficiently discriminate between them).

A first such step of comparing different copula models was taken in [12] where the authors evaluate designs for various copula choices against each other (in their Table 8). However, they have been using the same parameter values for all the copulas without considering the different meaning of the copula parameter for various copula families. Therefore, we instead provide in Table 4 an improved comparison between different dependence structures along the same Kendall's *τ* values by exploiting the relationship between the copula parameter to the measure of concordance *τ*. Thanks to this comparison, now the pure impact of the choice of the copula is highlighted. It turns out, that even in extreme case the efficiency losses are only small to moderate. They are greatest if Frank or Gumbel are used instead of Clayton, which may be explained by their opposing representations of tail dependencies.

**Table 4.** Losses in D-efficiency (in bold) by comparing the true copula model with the assumed one for a fixed Kendall's *τ*.

True Copula	Frank		Clayton		Gumbel	
Assumed Copula	Clayton	Gumbel	Frank	Gumbel	Frank	Clayton
τ=0.11	**2**.**24**	**0**.**67**	**1**.**99**	**2**.**70**	**0**.**82**	**2**.**75**
τ=0.45	**0**.**26**	**0**.**03**	**0**.**26**	**0**.**11**	**0**.**03**	**0**.**15**
τ=0.66	**1**.**09**	**0**.**11**	**1**.**04**	**1**.**28**	**0**.**14**	**1**.**57**
τ=0.76	**4**.**27**	**0**.**02**	**3**.**87**	**4**.**08**	**0**.**01**	**4**.**73**
τ=0.82	**8**.**24**	**0**.**01**	**10**.**91**	**10**.**96**	**0**.**01**	**8**.**43**

### 4.4. A more flexible model

Let us now allow the strength of the dependence itself be dependent upon the regressors *x*, a situation completely covered by our equivalence theorem. Thus here the copula parameters themselves become model-dependent such as, for example, in [28]. As in our context only positive associations (between efficacy and toxicity) make sense we consider in the following the *τ* modelled by a logistic:

(13) $$\tau (x,\alpha_{1})=\frac{e^{\alpha_{1}x}}{1+e^{\alpha_{1}x}},$$

which takes values in [0,1] for $\alpha_{1}\in [0,1]$.

Considering the Archimedian copulas Clayton $C_{1}$ and Gumbel $C_{2}$, the following relationships between the Kendall's *τ* and the copula parameters hold:

$$\begin{array}{rl} \tau_{C_{1}}(x,\alpha_{1}) & =\frac{\gamma_{1}(x,\alpha_{1})}{\gamma_{1}(x,\alpha_{1})+2}\;\mathrm{andso}\;\gamma_{1}(x,\alpha_{1})=\frac{2\tau_{C_{1}}(x,\alpha_{1})}{1-\tau_{C_{1}}(x,\alpha_{1})}\;\mathrm{for}\;\mathrm{the}\;\mathrm{Clayton}\;\mathrm{family};\\ \tau_{C_{2}}(x,\alpha_{1}) & =\frac{\gamma_{2}(x,\alpha_{1})-1}{\gamma_{2}(x,\alpha_{1})}\;\mathrm{andso}\;\gamma_{2}(x,\alpha_{1})=\frac{1}{1-\tau_{C_{2}}(x,\alpha_{1})}\;\mathrm{for}\;\mathrm{the}\;\mathrm{Gumbel}\;\mathrm{family}. \end{array}$$

Then, we model the current probability of success by a convex combination of the Clayton and the Gumbel copulas

$$C(\pi_{1},\pi_{2};\alpha_{1},\alpha_{2})=\alpha_{2}C_{1}(\pi_{1},\pi_{2};\gamma_{1}(x,\alpha_{1}))+(1-\alpha_{2})C_{2}(\pi_{1},\pi_{2};\gamma_{2}(x,\alpha_{1})),$$

and when we link them at the same *τ* values we end up with

$$C(\pi_{1},\pi_{2};\alpha_{1},\alpha_{2})=\alpha_{2}C_{1}(\pi_{1},\pi_{2};2\;e^{\alpha_{1}x})+(1-\alpha_{2})C_{2}(\pi_{1},\pi_{2};1+e^{\alpha_{1}x}).$$

The added flexibility of such a model is that the impact of the dependence structure and the association level is reflected by two different parameters. While the $\alpha_{2}$ parameter is strictly related to the structure of the dependence, the $\alpha_{1}$ parameter is only related to the measure of association Kendall's *τ*.

In Table 5 we report the efficiency losses with respect to the independence case (Product Copula). By fixing three localized values $\bar{\alpha_{1}}$, we assume three different intervals for *τ*. Corresponding to these intervals we fixed four localized values $\bar{\alpha_{2}}$. From the table it is clear that when the range of the *τ* increases, the losses in terms of D-efficiency can become quite substantial. By focusing on the various localized values for $\alpha_{2}$, it is evident that also the structure of the dependence plays a big role in the design obtained. Here, we can see that when the highest weight in the convex combination is on the Clayton copula, the efficiency losses are lowest.

**Table 5.** Losses in D-efficiency for the convex combination model in per cent (in bold).

	τ∈[0.5,0.95]	τ∈[0.5,0.99]	τ∈[0.5,0.995]
$\bar{\alpha_{2}}$	Loss in D-eff.	Loss in D-eff.	Loss in D-eff.
0.1	**11**.**15**	**31**.**97**	**47**.**22**
0.3	**6**.**51**	**25**.**11**	**40**.**33**
0.6	**2**.**50**	**17**.**20**	**32**.**27**
0.9	**1**.**11**	**10**.**90**	**24**.**96**

## 5. Discussion

In general, our theory forms the basis to investigate further showcase examples from the literature, like, for example, in [29] or eventually treat mixed discrete/continuous type models like in [30]. Particularly for the latter, but also quite generally the methods provided in this paper can thus be expected to be valuable for real applications from clinical trials, environmental sampling, industrial experiments, etc.

Although here we provide only examples on limited types of copulas, we might expect similar or greater effects for some more non-symmetric copulae (see eg. [31]), which are subject to our current investigations.

Note that in the convex combination example by focusing on $\alpha_{2}$ we could find designs with the sole purpose of efficiently discriminating between different copula models, which we plan to do future research on.

## Appendix

### Equivalence theorem

For all the basics in what follows cf.[16] For a given vector of parameters (β,α), let $M_{\left(\beta ,\alpha \right)}$ be the set of the information matrices generated as *ξ* ranges over the class of all set of probability distribution on X. Then $M_{\left(\beta ,\alpha \right)}$ is the convex hull of {m(x,β,α): x∈X}.

Let us now recall the definition of two derivatives that will play an important role in our theory.

Definition A ((Gâteaux and Fréchet derivative)). Considering two elements $M_{1}$ and $M_{2}$ in M, the *Gâteaux derivative of φ* at $M_{1}$ in the direction of $M_{2}$ is:

$$G_{\varphi }(M_{1},M_{2})=\lim_{\varepsilon \to 0^{+}}\frac{1}{\varepsilon }\{\varphi (M_{1}+\varepsilon M_{2})-\varphi (M_{1})\},$$

the *Fréchet derivative of φ* at $M_{1}$ in the direction of $M_{2}$ is:

$$F_{\varphi }(M_{1},M_{2})=\lim_{\varepsilon \to 0^{+}}\frac{1}{\varepsilon }\{\varphi \{(1-\varepsilon )M_{1}+\varepsilon M_{2}\}-\varphi (M_{1})\}.$$

The following are the properties of the derivatives that we defined before: the concavity of *φ* implies that

$$\frac{1}{\varepsilon }[\varphi \{(1-\varepsilon )M_{1}+\varepsilon M_{2}\}-\varphi (M_{1})]$$

is a non-increasing function of *ϵ* in 0<ε≤1. Hence when *φ* is concave, $F_{\varphi }(M_{1},M_{2})$ exists if we allow the value +∞.

It is clear that if we put ε=1 in the previous equation, we obtain: $F_{\varphi }(M_{1},M_{2})\geq \varphi (M_{2})+\varphi (M_{1})$.

According to the definitions of Fréchet and Gâteaux derivatives, we can stress the following relationship between them: $F_{\varphi }(M_{1},M_{2})=G_{\varphi }(M_{1},M_{2}-M_{1})$. Then, if we assume the differentiability of *φ* it is clear that for scalars $a_{i}$

$$F_{\varphi }\left(M_{1},\sum a_{i}M_{i}\right)=\sum a_{i}F_{\varphi }(M_{1},M_{i}).$$

Theorem A.2. Suppose to have a fixed parameters vector $(\bar{\beta },\bar{\alpha }),$ a concave function *φ* on $M_{\left(\bar{\beta },\bar{\alpha }\right)}$ which is also differentiable at all points of $M_{\left(\bar{\beta },\bar{\alpha }\right)}$ where φ(M)<−∞, so where a *φ* optimal measure exists. Then the following are equivalent:

- $\xi^{*}$ is *φ*-optimal;
- $F_{\varphi }(M(\xi^{*},\bar{\beta },\bar{\alpha }),M(\xi ,\bar{\beta },\bar{\alpha }))\leq 0,$ ∀ξ∈Ξ;
- $F_{\varphi }(M(\xi^{*},\bar{\beta },\bar{\alpha }),m(x,\bar{\beta },\bar{\alpha }))\leq 0,$ ∀x∈X;
- $\max_{x\in X}F_{\varphi }(M(\xi^{*},\bar{\beta },\bar{\alpha }),m(x,\bar{\beta },\bar{\alpha }))=\min_{\xi \in \Xi }\max_{x\in X}F_{\varphi }(M(\xi ,\bar{\beta },\bar{\alpha }),m(x,\bar{\beta },\bar{\alpha }))$.

Proof. Let us prove the theorem by double implications.

1. (i)⇒(ii) $\xi^{*}$ is *φ*-optimal. This means that $\varphi (M(\xi^{*},\bar{\beta },\bar{\alpha }))$ is maximal.
  For the properties of the function *φ*, the following relation holds:
  $$\varphi \{(1-\varepsilon )M(\xi^{*},\bar{\beta },\bar{\alpha })+\varepsilon M(\xi ,\bar{\beta },\bar{\alpha })\}-\varphi \{M(\xi^{*},\bar{\beta },\bar{\alpha })\}\leq 0$$
  for ε∈[0,1] and all ξ∈Ξ.
  For all the elements of $M_{\left(\bar{\beta },\bar{\alpha }\right)}$ holds that
  $$\left(1-\varepsilon )M(\xi^{*},\bar{\beta },\bar{\alpha })+\varepsilon M(\xi ,\bar{\beta },\bar{\alpha })=M\{(1-\varepsilon )\xi^{*}+\varepsilon \xi \right\}$$
  and this means, from the definition of the Fréchet derivative, that
  $$F_{\varphi }\{M(\xi^{*},\bar{\beta },\bar{\alpha }),M(\xi ,\bar{\beta },\bar{\alpha })\}\leq 0$$
  for all ξ∈Ξ.
2. (ii)⇒(iii) Since $m(x,\bar{\beta },\bar{\alpha })$ are elements of the convex hull $M_{\left(\bar{\beta },\bar{\alpha }\right)}$, the condition (iii) follows directly from the hypothesis.
3. (iii)⇒(iv) For a particular control *x* and the elemental informations *m*, since $F_{\varphi }\{M(\xi ,\bar{\beta },\bar{\alpha }),M(\xi ,\bar{\beta },\bar{\alpha })\}=0$, it must be that:
  $$\max_{x\in X}F_{\varphi }\{M(\xi ,\bar{\beta },\bar{\alpha }),m(x,\bar{\beta },\bar{\alpha })\}\geq 0$$
  But, according to the hypothesis, we have that for the design $\xi^{*}$
  $$\max_{x\in X}F_{\varphi }\{M(\xi^{*},\bar{\beta },\bar{\alpha }),m(x,\bar{\beta },\bar{\alpha })\}\leq 0.$$
  Hence
  $$\begin{array}{rl} & \max_{x\in X}F_{\varphi }\{M(\xi^{*},\bar{\beta },\bar{\alpha }),m(x,\bar{\beta },\bar{\alpha })\}=0\\ & \;=\min_{\xi }\max_{x\in X}F_{\varphi }\{M(\xi ,\bar{\beta },\bar{\alpha }),m(x,\bar{\beta },\bar{\alpha })\}. \end{array}$$
4. (iv)⇒(i) Suppose now that $\xi^{*}$ satisfies the hypothesis, then
  $$\max_{x\in X}F_{\varphi }\{M(\xi^{*},\bar{\beta },\bar{\alpha }),m(x,\bar{\beta },\bar{\alpha }))\}=0,$$
  that means that $F_{\varphi }\{M(\xi^{*},\bar{\beta },\bar{\alpha }),m(x,\bar{\beta },\bar{\alpha })\}\leq 0$, ∀x∈X. According to the definition of the matrices M∈M, any *M* can be written as $M(\xi ,\bar{\beta },\bar{\alpha })=\sum_{i=n}^{r}w_{i}m(x_{i},\bar{\beta },\bar{\alpha })$, where $\sum_{i=n}^{r}w_{i}=1$ and $w_{i}>0$ for every i=1,…,n.
  Then, since *φ* is differentiable at $M(\xi ,\bar{\beta },\bar{\alpha })$, it holds that:
  $$F_{\varphi }\{M(\xi^{*},\bar{\beta },\bar{\alpha }),M(\xi ,\bar{\beta },\bar{\alpha })\}=\sum_{i=1}^{n}\lambda_{i}F_{\varphi }\{M(\xi^{*},\bar{\beta },\bar{\alpha }),m(x,\bar{\beta },\bar{\alpha })\}\leq 0$$
  for every ξ∈Ξ.
  This means, clearly, that
  $$\varphi (M(\xi ,\bar{\beta },\bar{\alpha }))-\varphi (M(\xi^{*},\bar{\beta },\bar{\alpha }))\leq 0$$
  for every ξ∈Ξ, then $\xi^{*}$ is *φ*-optimal.

### D-optimality

Let consider now as design criterion the following function:

$$\varphi (M)=\left\{\begin{array}{ll} \log\det M & \text{if }M\text{ is non-singular}\\ -\infty & \mathrm{otherwise} \end{array}\right.$$

A design that maximizes such a *φ* function is called *D-optimal design*.

In the case of D-optimality the Fréchet and the Gâteaux derivatives have the following expression:

*Gâteaux derivative*

$$\begin{array}{rl} \log\det(M_{1}+\varepsilon M_{2})-\log\det M_{1} & =\log\det(I+\varepsilon M_{2}M_{1}^{-1})\\ & =\log\{1+\varepsilon \;\mathrm{tr}(M_{2}M_{1}^{-1})\}+O(\varepsilon^{2})=\varepsilon \;\mathrm{tr}(M_{2}M_{1}^{-1})+O(\varepsilon^{2}) \end{array}$$

Hence, $G_{\varphi }(M_{1},M_{2})=\mathrm{tr}(M_{2}M_{1}^{-1})$.

*Fréchet derivative*

$$\begin{array}{rl} F_{\varphi }(M_{1},M_{2}) & =G_{\varphi }(M_{1},M_{2}-M_{1})=\mathrm{tr}((M_{2}-M_{1})M_{1}^{-1})\\ & =\mathrm{tr}(M_{2}M_{1}^{-1}-I_{\left(k+l\right)})=\mathrm{tr}(M_{2}M_{1}^{-1})-(k+l) \end{array}$$

where (k+l) is the number of the model parameters.

We are ready now to give an equivalence theorem which holds in the particular case of the D-criterion.

Theorem A.3. For a fixed parameters vector $(\bar{\beta },\bar{\alpha }),$ the following properties are equivalent:

- $\xi^{*}$ is D-optimal;
- $\mathrm{tr}(M(\xi^{*},\bar{\beta },\bar{\alpha }{)}^{-1}m(x,\bar{\beta },\bar{\alpha }))\leq (k+l),$ ∀x∈X;
- $\xi^{*}$ minimize $\max_{x\in X}\mathrm{tr}(M(\xi^{*},\bar{\beta },\bar{\alpha }{)}^{-1}m(x,\bar{\beta },\bar{\alpha })),$ over all ξ∈Ξ.

Proof. The proof comes directly from the Theorem A.1 by imputing the Fréchet derivative for the D-criterion.
